# Advances in the discovery and study of *Trichoderma* natural products for biological control applications

**DOI:** 10.1039/d5np00017c

**Published:** 2025-06-06

**Authors:** Sophie Jin, Fabrizio Alberti

**Affiliations:** a Leibniz Institute for Natural Product Research and Infection Biology – Hans Knöll Institute (HKI) Beutenbergstraße 11a 07745 Jena Germany sophie.jin@leibniz-hki.de +49 3641 532-1740; b School of Life Sciences, University of Warwick Coventry CV4 7AL UK F.Alberti@warwick.ac.uk +44 (0) 24 765 23516

## Abstract

Covering: up to 2025

Reducing the prevalence of phytopathogens and their impact on crops is essential to reach sustainable agriculture goals. Synthetic pesticides have been commonly used to control crop disease but are now strongly linked to disease resistance, environmental pollution, depletion of soil biodiversity, and bioaccumulation, leading to adverse effects on human health. As a alternative, the prolific *Trichoderma* genus has been studied for its biocontrol properties, as well as its ability to promote plant growth and increase nutrient uptake. This is done through various mechanisms, one of which is the production of bioactive natural products with high chemical diversity. These include terpenoids, alkaloids, non-ribosomal peptides, polyketides and RiPPs. One of the most studied examples is 6-pentyl-2*H*-pyran-2-one, a volatile organic polyketide, which induces systemic acquired resistance, morphogenesis, and natural product biosynthesis in plants. Methods for culturing *Trichoderma* spp., isolating and characterising unique bioactive metabolites are discussed here, with an emphasis on dereplication strategies using metabolomics to optimise discovery. In addition, the role of genome mining for the study of natural product biosynthesis in *Trichoderma*, and more generally, filamentous fungi is discussed. Examples of bioinformatics tools available to date are listed here with applications in *Trichoderma* and other ascomycetes. New advances in genome engineering in *Trichoderma* are also detailed, providing insights into available strategies for the validation of biosynthetic gene clusters identified using genome mining. Finally, the use of a combination of omics approaches, namely metabologenomics, is presented as a growing field for natural product discovery in fungi.

## Abstract 2

*Trichoderma* spp. are talented producers of bioactive natural products, with applications in crop protection and growth promotion. We describe methods for the discovery of these compounds focused mainly on cultivation, genome mining and metabolomics.

## Graphical abstract

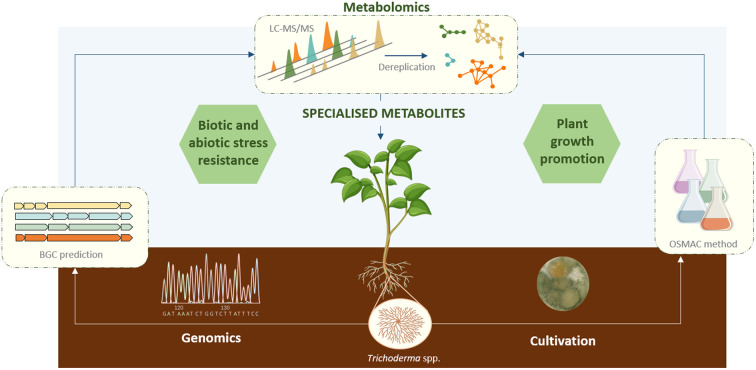

## 1 Introduction

Reducing the prevalence of phytopathogens and their impact on crops is essential to reach sustainable agriculture. Synthetic pesticides have been commonly used to control crop disease but are not sustainable. Indeed, they are linked to disease resistance, environmental pollution, depletion of soil biodiversity, and bioaccumulation linked to adverse effects on human health.^1,2^ Many chemical pesticides are now banned in the EU due to their concerning effects on environmental and human health.^3^ Instead, biocontrol agents (BCA) or biopesticides are getting increasing traction for their environmentally friendly characteristics. Examples of BCA include *Streptomyces griseoviridis* strain K61 (Mycostop®),^4^*Pythium oligandrum* strain M1/ATCC 38472 (Polyversum®, commercialised by De Sangosse), and *Trichoderma asperellum* strain TV1 (Xedavir®, commercialised by Xeda Italia S.r.l).

*Trichoderma* is a well-known genus of filamentous ascomycetes from the Hypocreaceae family and is widely distributed around the world, colonising a variety of ecological habitats such as soil, decaying wood, living plants (within the rizhosphere or as endophytes) and marine ecosystems.^5,6^ To date, the genus contains over 400 species spanning over several clades,^7^ of which the Harzianum clade includes most of the biocontrol agents used in agriculture.^8^*Trichoderma* spp. display biocontrol properties against plant pathogens through various mechanisms such as antibiosis, competition, or mycoparasitism.^9–12^ Aside from biotic stresses, *Trichoderma* spp. can also protect plants from abiotic stresses such as drought or salt stress *via* transcriptional activation of defence responses resulting in inhibition of seed germination and plant development, as well as stomatal regulation.^13–15^ In addition to biocontrol potential, *Trichoderma* spp. can also enhance plant development by improving nutrient uptake, photosynthesis and overall plant growth.^16–18^

## 2 *Trichoderma* spp. as a solution for crop management

One of the most used and researched genus of BCA is *Trichoderma* and a list of commercially available *Trichoderma*-based formulations can be found in the recent review by Martinez *et al.*^53^ and Woo *et al.*^54^ Species such as *T. asperellum*, *T. atroviride*, *T. gamsii*, *T. hamatum*, *T. polysporum*, *T. virens*, *T. viride* and *T. harzianum* are commercialised in Europe, the US, Canada, Australia, New Zealand, South Africa, Vietnam and India as biological control agents. One example of product available in Europe is ASPERELLO® T34 Biocontrol (*Trichoderma asperellum* T34), a fungicide sold as a powder to be mixed with the substrate before transplantation.^54^ Another strain which is available in Europe and the US is *T. harzianum* Rifai T-22 (T-22™ HC, BioWorks®), a formulated preventative fungicide which can be applied to seeds, soil and other propagative parts, protecting the plants from root pathogens. Other products utilising *T. harzianum* include AkTRIvator® (Canna International BV, Breda, the Netherlands) Trichosan® (Vitalin Pflanzengesundheit GmbH, Ober-Ramstadt, Germany) and Promot® WP (JH Biotech Inc., Ventura, California).

Many *Trichoderma* species exhibit an antagonistic effect on plant pathogens through antibiosis, competition or mycoparasitism^11^ and can thus improve productivity,^10,55^ and this review will focus mainly on antibiosis as a mode of action for plant disease suppression. Antibiosis is a common phenomenon seen in *Trichoderma* spp. and can be defined as the interaction between microorganisms through the production of specialised metabolites and resulting in toxicity or growth inhibition for one of the microorganism.^56^ Many recent studies have explored the potential of *Trichoderma* spp. as biocontrol agents against pathogenic fungi and bacteria of plants^34,35,38,43,45,50,51^ and the subject has been extensively reviewed.^54,57–59^ Examples of species of *Trichoderma* which have shown antagonism against plant pathogens and their mechanisms are detailed in Table 1. An exhaustive list of interactions between *Trichoderma* spp. and plants can be found in the review by Sood *et al.*^59^ Reports of *Trichoderma* spp. as an insect pests BCA have also been reviewed by Poveda *et al.*^60^

**Table 1:** List of *Trichoderma* species exhibiting antifungal and/or antibacterial activity against plant pathogens from recent studies

Species	Mode of action	Pathogens	Reference
*Trichoderma viride*	Specialised metabolites and volatile organic compounds (VOCs)	*Fusarium oxysporum*, *Pythium aphanidermatum*, *Rhizoctonia solani*, *Sclerotium rolfsii*, *Candida albicans*, *Pythium ultimum*, *Nigrospora oryzae*	19–21
*Trichoderma atroviride*	Cell wall degrading enzymes, antibiosis (polyketides against *R. solani*), VOCs	*Verticillium dahlia*, *Rhizoctonia solani*, *Botrytis cinerea*, *Phytophthora capsica*, *Plasmopara viticola*, *Nigrospora oryzae*	21–25
*Trichoderma harzianum*	Cell wall degrading enzymes, VOCs, specialised metabolites (gliotoxin, peptaibols, antrhaquinones, methyl dihydrojasmonate)	*Sclerotinia sclerotiorum*, *Rhizoctonia solani*, *Plasmopara viticola*, *Pythium aphanidermatum*, *Fusarium oxysporum*	19 and 25–29
*Trichoderma koningii*	Parasitism and specialised metabolites: trichokonins	*Sclerotinia sclerotiorum*	30 and 31
*Trichoderma pseudokoningii*	Specialised metabolites: peptaibols and trichokonins	*Fusarium oxysporum*	32
*Trichoderma koningiopsis*	Specialised metabolites: trichodermin, azetidine, 2-phenylethanol, and ethyl hexadecanoate, polyketides, VOCs	*Pyricularia oryzae*, *Aspergillus fumigatus*, *Botrytis cinera*, *Colletotrichum gloeosporioides*, *Fusarium oxysporum*, *Verticillium dahliae*	33–37
*Trichoderma asperellum*	VOCs, chitinases	*Fusarium incarnatum*, *Plasmopara viticola*, *Nigrospora oryzae*	21, 25 and 38–40
*Trichoderma virens*	Gliotoxin and trichodermamides	*Rhizoctonia solani*	23 and 41
*Trichoderma longibrachiatum*	Parasitism through cell wall degrading enzymes against nematodes, peptaibol production	*Magnaporthiopsis maydis*, *Meloidogyne incognita*, *Heterodera avenae*, *Pseudomonas syringae*	42–44
*Trichoderma reesei*	Cell wall degrading enzymes and secretion of phenols, and antifungal compounds (peptaibols, anthocyanins, β-caryophyllens)	*Rhizoctonia solani*, *Fusarium oxysporum*	23 and 45
*Trichoderma lignorum*	Specialised metabolites: gliotoxin	*Rhizoctonia solani*, *Sclerotinia americana*	46
*Trichoderma brevicompactum*	Cell wall degrading enzymes, secretion of indole acetic acid, trichodermin	*Fusarium oxysporum*	47 and 48
*Trichoderma carraovejensis*	Uncharacterised antagonism	*Phaeoacremonium minimum*, *Phaeomoniella chlamydospora*, *Diplodia seriata*	49
*Trichoderma* spp.	Spore adhesion and niche exclusion, stimulating gene expression involved in plant-disease resistance, (±)-trichodermatrione A production	*Phaeoacremonium minimum*, *Fusarium oxysporum*, *Xanthomonas oryzae*	50–52

## 3 Specialised metabolism of *Trichoderma* spp.

*Trichoderma* species are talented producers of specialised metabolites. With over 200 isolated compounds reported recently, the potential of *Trichoderma* spp. still remains untapped.^61,62^ Not only are those compounds chemically diverse, but they also exhibit a broad spectrum of bioactivity, with a wide range of applications. The vast majority of these specialised metabolites are synthesised by biosynthetic gene clusters (BGC). Genes in a BGC encode for specific steps in the biosynthesis pathway of metabolites and are co-localised in the genome, which is thought to be due to evolutionary pressure (mainly coinheritance and coregulation).^63^ In fungi, a typical BGC contains genes encoding one or more core enzymes which catalyse the synthesis of the backbone of the final product, several accessory enzymes (hydrolases, epimerases, oxidoreductases, and methyltransferases amongst others) which further modify the backbone, regulatory proteins, transport-related proteins and in some cases, proteins related to resistance mechanisms.^64^

### 3.1 Chemical diversity of natural products in *Trichoderma* spp.

**Fig. 1:**
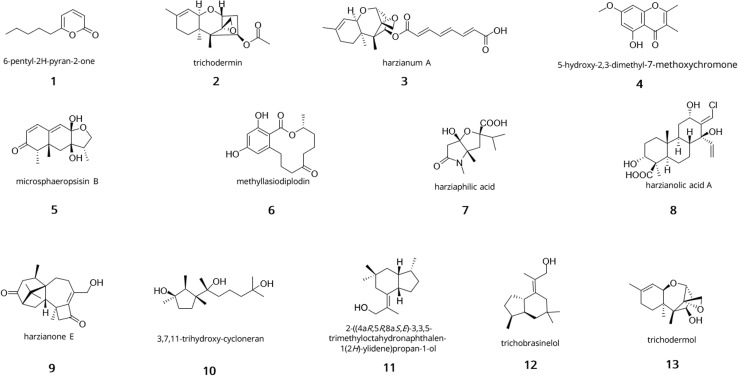
Snapshot of the chemical diversity of terpenoids and polyketides from *Trichoderma*. 6-Pentyl-2*H*-pyran-2-one (1); trichodermin (2); harzianum A (3); 5-hydroxy-2,3-dimethyl-7-methoxychromone (4); microsphaeropsisin B (5); methyllasiodiplodin (6); harziaphilic acid (7); harzianolic acid A (8); harzianone E (9); 3,7,11-trihydroxy-cycloneran (10); 2-((4*aR*,5*R*,8*aS*,*E*)-3,3,5-trimethyloctahydronaphthalen-1(2*H*)-ylidene)propan-1-ol (11); trichobrasinelol (12); trichodermol (13).

Reviews of specialised metabolites from the *Trichoderma* genus have been published in 2016,^65^ 2021 ^62^ and most recently in 2023.^66^ The most recent review on specialised metabolites from *Trichoderma* spp. focused on marine strains and listed the isolation of 445 specialised metabolites over the past 30 years, some of which presented new carbon skeletons.^67^ As this subject has been extensively investigated, this section will focus on the most relevant natural product classes with a discussion on some of their corresponding examples. Structures of each example can be found in Fig. 1.

#### 3.1.1 Volatile organic compounds and terpenoids

In the context of biocontrol, volatile organic compounds (VOCs) represent one of the major classes of specialised metabolites from *Trichoderma* as many play a role in modulating plant defence response against biotic and abiotic stresses. VOCs are low molecular weight molecules with low boiling point, high hydrophobicity and usually a fragrant odour.^68^ An extensive review of VOCs from *Trichoderma* has recently been published by Jiménez *et al.*,^69^ and detailed their involvement in plant growth and pathogen defence response. One of the most potent and widespread volatiles is the polyketide 6-pentyl-2*H*-pyran-2-one (6-PP, 1). It was found in many species of *Trichoderma*, notably *T. harzianum*, *T. viride*, *T. atroviride*, *T. koningii*, *T. asperellum*, *T. longibrachiatum*, and *T. pseudokoningii*.^59^ It has been described as an antifungal,^70–73^ an elicitor of systemic acquired resistance mechanisms and morphogenesis as well as an activator of antimicrobial natural product biosynthesis in plants.^74,75^ Other volatiles include monoterpenes, sesquiterpenes, small alkenes, alcohols and ketones.^69^

In the class of terpenoids, trichothecenes and their derivatives are mycotoxins which have been reported in many *Trichoderma* species like *T. brevicompactum*, *T. harzianum*, *T. viride*, *T. longibrachiatum*, *T. atroviride*, *T. erinaceum*, *T. citrinoviride*^76^ and more broadly in species of *Fusarium*, *Cryptomela*, *Spicellum*, *Myrothecium*, *Stachybotyrs*, *Cephalosporium* and *Trichothecium*.^77^ Trichothecenes are sesquiterpene epoxides with a tricyclic 12,13-epoxytrichothec-9-ene (EPT) core and differ from the substitution found on the EPT, classifying them into 4 types (A, B, C, D).^78^ While the epoxide is essential for bioactivity, the nature of the activity can vary based on the substitutions found on the EPT.^79,80^ For example, trichodermin (2) inhibits protein synthesis whereas harzianum A (3) acts as a powerful herbicide.^81–84^ The biosynthetic gene cluster (TRI) for both compounds has been established in *Fusarium* species^85^ and orthologs were found in *Trichoderma*.^86^ Briefly, the gene cluster for trichothecene biosynthesis in *Fusarium* species is composed of 15 genes, spread over 3 chromosomes, and is initiated by the trichodiene synthase Tri5, which catalyses the cyclisation of farnesyl pyrophosphate to trichodiene. Tri1, Tri3, Tri4, Tri8, Tri11, and Tri10 catalyse the other steps of the biosynthesis.^87–89^

A comprehensive list of other terpenoids produced by *Trichoderma* can be found in the reviews by Zhang *et al.*,^62^ and most recently by Bai *et al.*^90^ and Guo *et al.*^66^

#### 3.1.2 Alkaloids and peptides

**Fig. 2:**
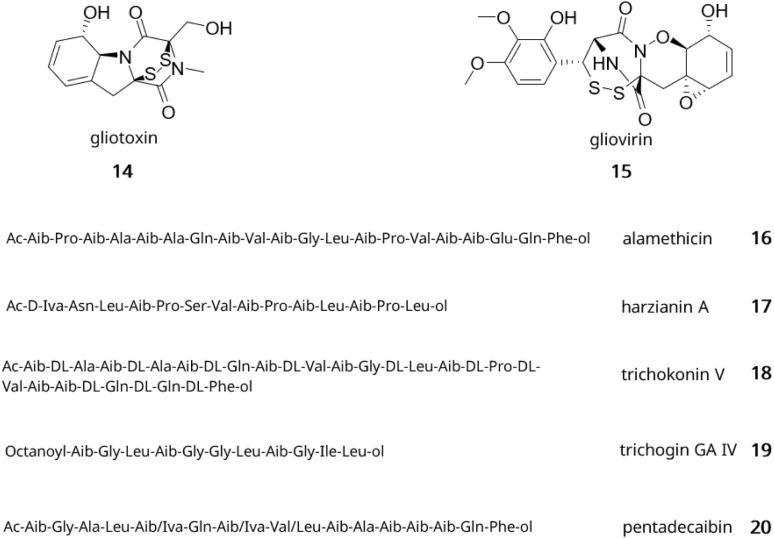
Examples of alkaloids and peptides from *Trichoderma*. Gliotoxin (14); gliovirin (15); alamethicin (16); harzianin A (17); trichokonin V (18); trichogin GA IV (19); pentadecaibin (20).

Most nitrogen-containing SMs found in *Trichoderma* are alkaloids and non-ribosomal peptides. Examples of alkaloids with bioactivity include epipolythiodioxopiperazines (ETP) and other specialised metabolites containing a diketopiperazine ring. The two toxins gliotoxin (14) and gliovirin (15) are two well-known examples of ETP with strong antimicrobial properties^91^ (Fig. 2). The biosynthesis of gliotoxin was first elucidated in *Aspergillus fumigatus* and was found to depend on 13 genes,^92^ with GliP encoding an essential non-ribosomal peptide synthetase (NRPS) responsible for the formation of the diketopiperazine scaffold.^93^ A list of other alkaloids and their bioactivities can be found in the review by Bai *et al.*^90^

**Fig. 3:**
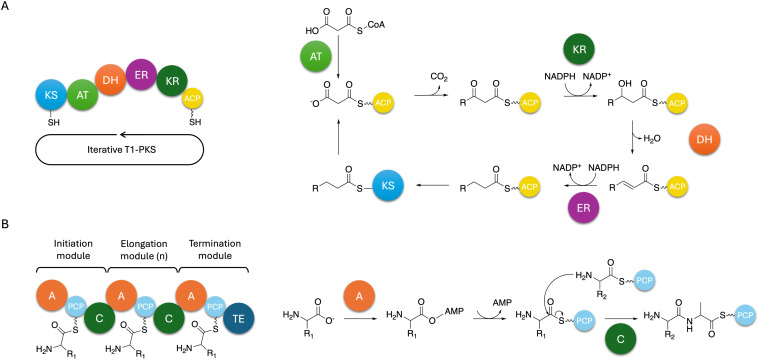
Mechanism of type I iterative PKS and NRPS in fungi. (A) A type I iterative PKS is composed of the essential ketosynthase (KS), acyltransferase (AT), and thioesterase (TE) domains. Additional domains include ketoreductase (KR), enoylreductase (ER) and dehydratase (DH) domains. The biosynthesis starts by the transthiolesterification of an acyl-CoA on the active site thiol of the KS. In parallel, a malonyl-CoA unit is also transferred to the thiol of the phosphopantetheine (PP) prosthetic group of the acyl carrier protein (ACP) group. These two reactions can often be catalysed by the AT. The KS catalyses decarboxylative Claisen condensation to form a β-ketoacyl thioester. The KR then reduces the keto group at the β-carbon into a hydroxyl, the DH further catalyses the formation of a double bond by dehydration of the hydroxyl group, and the ER reduces the double bond. The chain can then be loaded back onto the active site thiol of the KS, and the cycle can continue to then finally be released by the TE domain in the case of non-reducing and partially-reducing PKSs, and a *trans*-acting hydrolase in the case of highly-reducing PKSs. (B) An NRPS is composed of repeating units of adenylation (A), peptidyl carrier protein (PCP), and condensation (C) domains organised in modules. The number of elongation modules can vary and will dictate the size of the final peptide. The biosynthesis starts with the activation of an amino acid catalysed by the A domain. The A domain binding site sequence will dictate the amino acid to be incorporated into the peptide chain. The activated amino acid is then transferred to the thiol unit of the PP group of the PCP. The C domain then catalyses the formation of a peptide bond between this amino acid and the subsequent amino acid attached to the PCP of the next module, and so on until reaching the termination module. The TE domain then catalyses the release of the peptide.

NRPSs are multi-domain enzymes with a multi-modular architecture described in Fig. 3. NRPSs are composed of an adenylation (A), a condensation (C), a peptidyl carrier protein (PCP) and a thioesterase (TE) domain organised in modules where each module usually contains one instance of those domains. Despite this modular organisation, it is still challenging to predict the structure of these peptides due to module skipping.^94^ Indeed, it was reported that Tex2, an NRPS from *T. virens*, produces two classes of peptaibols with either 11 or 14 residues.^94^

In *Trichoderma*, peptide specialised metabolites are predominantly peptaibols. These peptides range from 5–20 amino acids and are characterised by their high content of 2-aminoisobutyric acid, their acetylated (peptaibols) or acylated (peptaibiotics) N-terminus and their C-terminal amino alcohol.^95^ Some examples of peptaibols include alamethicin (16), harzianins (17), trichokonins (18), and lipopeptaibols like trichogin GA IV (19) as shown in Fig. 2. Their antimicrobial activity has been linked to their helical structure and amphipathic nature. Due to those properties, they can form ion channels in the lipid membranes causing permeabilisation of the cells.^96,97^ Over 440 peptaibol sequences have been reported and databases of peptaibols and peptaibiotics containing details of their biological source, activity, 3D structure and accompanying bibliographical data can be accessed or downloaded for research purposes.^97–100^

Another class of peptides include ribosomally synthesised and post-translationally modified peptides (RiPP) but scarce research is found on that class of specialised metabolites in *Trichoderma*. What is known is through genome mining using tools like RIPPMiner,^101^ RRE-Finder^102^ or RiPPER^103^ and rely heavily on known BGC structures of RiPPs like ustiloxins.^104^ This limits the number of identifiable RiPPs as novel classes of RiPPs can be missed. Recently, a combined genomic and transcriptomics approach along with careful manual curation enabled the discovery of a series of potential new RiPP gene clusters in *Trichoderma*, missed by other traditional methods like antiSMASH.^105,106^

#### 3.1.3 Polyketides

Polyketides are a class of specialised metabolites with high structural diversity and vast range of bioactivity.^107^ They represent one of the major types of specialised metabolites in *Trichoderma*, with over 20 predicted BGCs per genome on average, second after NRPS BGCs.^108^ Polyketides are assembled by polyketide synthases (PKS) through multiple rounds of decarboxylative Claisen condensation reactions (Fig. 3). PKSs are multi-domain enzymes containing acyltransferase (AT), ketosynthase (KS) and thioesterase (TE) units for non-reducing and partially-reducing PKSs.^109^ In the case of highly-reducing PKSs, the release is catalysed by a *trans*-acting hydrolase, acyl transferase or in rare occasions a PLP-dependent domain.^110^

In fungi, polyketides are classified in two categories: aromatic and aliphatic compounds, due to the domain structure of fungal PKSs.^111^ These structures range from non-reducing (no reductive steps) to highly reducing (varied levels of reduction) based on their domain composition. Most known fungal PKSs fall into the type I iterative PKSs and resemble mammalian fatty acid synthases^112^ but type III PKSs also exist.^113,114^ In type I iterative PKSs, the same module is used over cycles of elongation to produce the final product as opposed to bacterial type I PKSs. Most recently, a review on highly reducing fungal PKSs (hr-PKSs) was published by Cox^110^ detailing the catalytic activities of each domain involved in hr-PKS biosynthesis as well as their stereoselectivity.

**Fig. 4:**
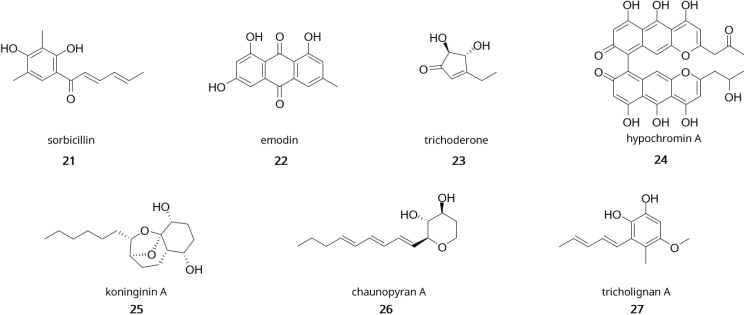
Examples of polyketides from *Trichoderma*. sorbicillin (21); emodin (22); trichoderone (23); hypochromin (24); koninginin A (25); chaunopyran A (26); tricholignan A (27).

Examples of polyketides from *Trichoderma* are shown in Fig. 4 and include sorbicillinoids (sorbicillin 21), anthraquinones (emodin 22), cyclopentones (trichoderone 23), naphthopyrones (hypochromin A 24) and koninginins (koninginin A 25).^67^ Sorbicillinoids have been isolated in many species of *Trichoderma* and are responsible for the yellow pigmentation observed in cultures. They hold a wide range of bioactivity including antioxidant^115^ and antimicrobial.^116^ The gene cluster for their biosynthesis has been described in *T. reesei* and contains 8 genes, two of them being transcription regulators.^117,118^ Briefly, Sor1 forms the polyketide chain, which is then further elongated and methylated by Sor2. The released aldehyde undergoes spontaneous cylcisation to form sorbicillin. The rest of the tailoring enzymes like Sor3 (FAD-dependent monooxygenase) and Sor4 (FAD/flavin mononucleotide-containing dehydrogenase) are responsible for the formation of the key intermediate sorbicillinol.^119^ Emodin (22) corresponds to a type of anthraquinone from *Trichoderma* with antifungal, antibacterial, and antioxidative properties which have been linked to the antagonistic activity against phytopathogens.^120–122^ Koninginins were found in multiple species of *Trichoderma*, most notably in *T. koningii* where the first koninginin (koninginin A 25) was isolated,^123^ which displayed cytotxic activity and plant growth regulating properties.^123,124^

### 3.2 Traditional methods for natural product discovery and examples in *Trichoderma* spp.

For the longest time, bioactive natural products were discovered in cultivable microorganisms using activity-guided methods, for which penicillin is a prime example.^125^ However, due to the complexity of the environments harbouring those microorganisms, the number of specialised metabolites which can be isolated from microbial cultures is limited. One traditional approach to promote the isolation of new natural products is the “One Strain Many Compounds” (OSMAC) technique, a concept that has been first introduced by Schiewe *et al.*,^126^ 25 years ago.

#### 3.2.1 “One strain many compounds”: an effective approach for unlocking metabolic pathways

The concept of OSMAC is to modulate culture conditions to induce production of new specialised metabolites. These changes include modification of temperature, pH, or nutrient sources. This method has been demonstrated in multiple species of fungi, leading to the discovery of new metabolites.^127^ Indeed, when *Penicillium* sp. F23-2 was grown in a shaking incubator in YPG medium, 5 new sorbicillinoids were discovered, namely, sorbicillamines A–E.^128^ In *Dothideomycete* sp. CRI7, 6 polyketides were isolated and characterised, 2 of them showing cytotoxicity.^129^ Similar studies in *Hypomontagnella monticulosa* Zg15SU yielded a new briarian diterpene named brianthein W, which showed anticancer activity.^130^ In *Aspergillus carneus*, the addition of sea salt to modified Czapeck medium lead to the isolation of 3 new compounds, namely, isopropylchaetominine, isoterrelumamide A and 5′-*epi*-averufanin.^131^ Finally, in *Trichoderma harzianum* M10, by modulating the culture conditions, the new compound 5-hydroxy-2,3-dimethyl-7-methoxychromone (4) was isolated and demonstrated antifungal activity against *R. solani*.^132^ More instances of new specialised metabolites using OSMAC in fungi can be found in the review by Pan *et al.*,^133^ and Pinedo *et al.*^134^

However, the traditional OSMAC approach has limitations due to low ability to reproduce the natural habitats of isolates within laboratory settings. This method has then included other techniques such as co-cultures and epigenetic modifications to circumvent those limitations.

In the first instance, co-cultivation of *Trichoderma* spp. with either bacteria or fungi has proven to induce production of metabolites as demonstrated by the discovery of two new sesquiterpenes microsphaeropsisin B/C (5) along with two new methyllasiodiplodins (6) from co-cultures of *Trichoderma* sp. strain 307 and *Acenitobacter jonhsonii* strain B2.^135^ Similarly, co-cultures of *T. atroviride* SG343 and *B. subtilis* 22 were found to inhibit the growth of *Fusarium graminearum*, when monocultures failed to show any antifungal activity.^136^ Similar findings were observed in co-cultures of *B. amyloliquefaciens* ACCC11060 and *T. asperellum* GDFS1009, where increase antimicrobial production was detected compared to monocultures.^137^ Fungal–fungal co-cultures have also displayed their potential for natural product discovery in *Trichoderma* spp. Indeed, co-cultivation of *T. harzianum* M10 with *Talaromyces pinophilus* F36CF was found to induce production of harziaphilic acid (7),^138^ whereas co-cultivation of *Chaunopycnis* sp. (CMB-MF028) and *T. hamatum* (CMB-MF030) activated production of chaunopyran A (26), identified as a broad-spectrum antifungal.^139^ More recently, efforts have been directed at optimisation of *Trichoderma* consortia for plant growth promotion as exemplified in studies on cucumber plants.^140,141^ In one study, researchers found that simultenaous inoculation of *Trichoderma* strains promoted better growth of cucumber seedlings and fermentation of 96 h to 120 h yielded the best production of growth promoting metabolites.^140^ Another study focused on optimising the combination of *Trichoderma* strains and found that co-cultures with *T. asperellum* GDSF1009, *T. asperelloides* Z4-1, *T. harzianum* 10 569, and *T. asperellum* 10 264 yielded the best results for both seed germination and antagonistic activity against *F. oxysporum*.^141^

The second one relies on epigenetic modification as its role in regulating gene expression involved in secondary metabolism is well-known and was proven for the first time in *Aspergillus*.^142–144^ Indeed it was shown that deletion of a histone deacetylase (HDAC) resulted in overexpression of three biosynthetic gene clusters involved in the synthesis of penicillin, sterigmatocystin and terraquinone A.^142^ However, examples of the use of chemical epigenetic modifiers in *Trichoderma* spp. for natural product discovery remain scarce.^145,146^ In *T. harzianum* XS-20090075, the use of 10 μM sodium butyrate, a known histone deacetylase inhibitor, induced expression of genes involved in terpenoid biosynthesis and led to the isolation of harzianolic acid A (8), harzianone E (9), and 3,7,11-trihydroxy-cycloneran (10).^146^ In *T. atroviride*, application of the histone deacetylase inhibitor TSA1 induced specialised metabolism, with expression of genes related to peptaibol and terpene biosynthesis and linked to increased inhibitory activity against *R. solani*.^145^

Another method is ribosome engineering, which relies on the appearance of random mutations in RNA polymerase or ribosomes when organisms are exposed to antibiotics which target ribosomes. This approach has been proven to increase yield and even enable the production of new specialised metabolites. It has been used successfully in Actinomycetes with the activation of cryptic BGCs in *Streptomyces* species, amongst others.^147–150^ However, in fungi, examples of this approach have only been reported in *Penicillium* and *Aspergillus* species. In *Penicillium purpurogenum* G59, gentamycin induced the production of janthinone, fructigenine A, aspterric acid methyl ester and citrinin, while neomycin induced the production of curvularin, citrinin, penicitrinone A, erythro-23-*O*-methylneocyclocitrinol and 22*E*-7α-methoxy-5α, 6α-epoxyergosta-8(14),22-dien-3β-ol in selected resistant mutants.^151,152^ In *Aspergillus versicolor* ZBY-3, neomycin-resistant mutants were shown to produce six peptides with antitumor activity, absent in the wild type extracts.^153^ Most recently, a study in *Actinomadura* sp. used random mutagenesis methods including ribosome engineering with streptomycin to increase the yield of pentostatin, an antitumor drug, by close to 34%.^154^

#### 3.2.2 Importance of dereplication

While OSMAC approaches and new cultivation systems like iChip have enabled the discovery of new compounds, this was only achieved by exploiting metabolomics and dereplication techniques. Indeed, dereplication is essential to ensure purification efforts are not directed towards the re-discovery of known compounds. Currently, this is mostly done using ultra high-pressure chromatography (UHPLC), high resolution mass spectrometry (HR-MS), HR-MS/MS, and nuclear magnetic resonance (NMR) spectroscopy, then linking it to existing databases like the Dictionary of Natural Products,^155^ the Natural Product Atlas,^156^ REAXYS®, METLIN,^157^ COCONUT^158^ and many others. A full list of all databases available up to date can be found in the review by Sorokina *et al.*^159^ This subject has now been extensively reviewed^160–164^ and most recently by Gaudêncio *et al.*^165^ Dereplication softwares have been constructed to facilitate the processing of the increasingly large amount of spectral data being generated. One well-known platform is the Global Natural Products Social Molecular Networking (GNPS) which uses MS/MS fragmentation data to group compounds based on structure similarity and searches available databases for corresponding natural products.^166^ A newer tool for molecular networking developed in 2019 is SIRIUS 4.^167^ This software establishes the molecular fingerprint of any known or unknown compound from its multi-level fragmentation and mass spectrum, to then link it to the molecular fingerprint of any compound from public databases like PubChem, leading to structural prediction. Authors report a rate of over 70% correct structure identification on two metabolomics datasets of skin and faecal origin.^167^ The use of NMR spectroscopy has now also been described for dereplication.^165^ MADByTE uses 2D NMR spectra to identify structural similarities between compounds within complex mixtures. This study showed the application of MADByTE on fungal natural products and demonstrated its potential with the discovery of three new palmarumycins.^168^ However, no reports of this method were found applied to *Trichoderma* spp.

## 4 The role of omics in the study of specialised metabolites biosynthesis in *Trichoderma* spp.

Along with analytical chemistry techniques, the advent of next generation sequencing has enabled the use of genome mining as a tool for dereplication. Using genome sequences to predict BGCs and prioritise candidates with unknown products has recently been the driving force for natural product discovery.^169^

### 4.1 Sequencing efforts towards *Trichoderma* genomes

Efforts in isolation and identification of *Trichoderma* species are continuously reported on the International Commission on *Trichoderma* Taxonomy (ICTT) platform.^170^ Guidelines for molecular taxonomy of *Trichoderma* were established in 2020 by Cai *et al.*,^170^ and an inventory of over 450 unique species was drawn out. Methods for identification now focus on DNA-based techniques like barcoding.^170,171^ In 2008, the Joint Genome Institute (JGI) published the first full genome sequence for *Trichoderma reesei*.^172^ This major achievement paved the way for many other species of *Trichoderma* to follow.^173,174^ Now, over 150 genomes of *Trichoderma* can be found on NCBI, and 9 reference genomes are available as RefSeq annotations.^175^ These annotations include *T. reesei*, *T. atroviride*, *T. asperellum*, *T. breve*, *T. harzianum*, *T. aggressivum*, *T. virens*, *T. citrinoviride*, and *T. gamsii*. More efforts by the JGI community are underway to increase the number of available *Trichoderma* genomes.^173^ In a comparative genomic study on 12 species of *Trichoderma*, Kubicek *et al.*^108^ found that the number of PKS BGCs are similar to the numbers in *Aspergillus* spp. but that *Aspergillus* NRPS and terpenoid BGCs are significantly outnumbered by the ones in *Trichoderma*. However, the relationship between those BGCs and their metabolites remains elusive.

### 4.2 Advances in genome mining tools and applications in *Trichoderma* spp.

The number of tools for automated BGC prediction and analysis based on genomic data has been increasing ever since the first iteration of antiSMASH in 2011,^176^ now reaching its 7th version.^177^ This pipeline uses a rule-based approach to find BGCs in bacteria, fungi and plant genomes based on available information on BGCs in public databases like MiBIG.^178^ AntiSMASH can now identify up to 81 cluster types with its latest update, which also includes improvements in substrate specificity for PKS and NRPS genes, prediction of RiPP clusters, and prediction of transcription factor binding sites. Following on this work, other tools were developed like PRISM, MIDDAS-M and DeepBGC for BGC detection;^179–181^ BiG-SCAPE/CORASON, Big-SLICE, MultiGeneBlast, and EvoMining for BGC clustering and phylogenetic analyses.^182–185^ The amount of interest towards gene cluster families (GCF) based research led to the generation of BiG-FAM, a dedicated database created in 2021.^185^ Recent reviews on bioinformatics tools for BGC mining are numerous^106,169,186–191^ with the most recent one by Cano-Prieto *et al.*^192^

Tools specific for fungal natural products mining like TOUCAN,^193^ FunOrder,^194^ CO-OCCUR,^195^ CLOCI^196^ and FunGeneClusterS^197^ have also been reported. The first software, alike to DeepBGC, uses supervised learning to predict BGCs but uses amino acid sequences to do so. Compared to fungiSMASH, DeepBGC, TOUCAN provided better performance in BGC prediction and identification of core enzymes in both *A. niger* and *A. nidulans*.^193^ FunOrder performs co-evolution analysis to identify essential genes in BGCs and prioritises them for further studies. Using CO-OCCUR, Gluck *et al.*^195^ found over 3000 putative BGC and 719 unique GCFs within the *Dothideomycetes* fungal lineage using co-occurrence frequency of gene pairs. Of those, known BGCs and their respective compounds include aflatoxin-like dothistromin,^198^ dimethylcoprogen,^199^ alternapyrone,^200^ and chaetoglobosins.^201^ Similarly to FunOrder, CLOCI identified gene clusters based on co-evolution, outperforming antiSMASH.^196^ Lastly, FunGeneClusterS uses a combination of genomic and transcriptomic data to predict BGC based on co-regulation patterns.^197^ Using transcriptomics to guide natural product discovery has been done in *A. niger*. Indeed, based on 283 transcriptomes, Kwon *et al.*^202^ generated co-expression networks and identified six transcription factors which regulate specialised metabolism in *A. niger*. The metabolome of the strains overexpressing those transcription factors displayed over 140 more metabolites compared to the control strain, some of which were associated with known gene clusters such as the alkylcitrates BGC.^202,203^

In *Trichoderma*, genome mining has uncovered new specialised metabolites with great chemical diversity including polyketides, terpenes, non-ribosomal peptides, and RiPPs. Indeed, Yan *et al.*^204^ found a novel class of hybrid polyketides with a terpene-like structure and a d-glucose esterified core in *Trichoderma afroharzianum* T-22, namely treconorin (28). In the same fungus, genome mining of PKS-NRPS hybrid clusters combined with heterologous expression in *A. nidulans* yielded six new tetronate SMs, trihazones A–F (29).^205^ In *T. viride*, genome mining enabled the isolation of a novel 5/6 bicyclic sesquiterpene (Fig. 1, 11) and its esterified derivative.^206^ In the class of non-ribosomal peptides, genome sequencing of *Trichoderma* spp. MMS1255 led to the discovery of a set of 5 unique 15-residue peptaibols, subsequently named pentadecaibins I–V (20).^207^ Finally, using whole-genome sequencing and transcriptomics, Vignolle *et al.*^106^ developed a new approach for mining RiPP clusters which revealed over 600 potential RiPP BGCs across 4 *Trichoderma* genomes. This work still requires molecular validation to confirm the accuracy of the framework.

### 4.3 Innovations in resistance-guided genome mining in fungi

Another approach to mine for bioactive natural products is to exploit the presence of resistance genes.^208,209^ One of the first examples of antibiotic discovery using self-resistance-based genome mining was the discovery of a set of thiotetronic acid derivatives from *Salinispora* bacteria.^210^ Research on this topic has historically been applied to bacteria but growing interest has been shown to fungi recently. One of the first pioneering works in this field was done in *Penicillium brevicompactum*. By looking for a BGC containing a IMPDH homolog (target of mycophenolic acid), a 25-kb BGC was identified and linked to the biosynthesis of mycophenolic acid.^211^ Work in *A. nidulans* followed, where the presence of a proteasome inhibitor (inpE) in a cryptic BGC was hypothesised to be involved in self-resistance. Using serial promoter exchanges to activate the cluster, the elucidation of fellutamide B biosynthesis^212^ was achieved. These findings illustrated the potential of resistance-guided genome mining in fungi. Following this study, the same approach was applied to *T. afroharzianum* to elucidate the biosynthesis of harzianic acid, where a acetohydroxyacid synthase homolog was found within the BGC and proven to be a target of harzianic acid.^213^ Similarly, the BGC for restricticin in *A. nomius* was identified based on co-localisation of its target, a lanosterol 14α-demethylase (CYP51) paralog, displaying unique mutations and less susceptible to inhibition.^214^

Subsequently, more automated methods were used to facilitate discovery. Currently, 3 frameworks exist for computer-aided discovery using resistance genes in fungi. In 2019, the FRIGG pipeline was developed based on 50 *Aspergillus* genomes and was able to predict over 70 unique clusters containing putative resistance genes. It was further validated with the correct identification of the previously characterised fellutamide B cluster.^215^ Later on, Jenkinson *et al.*^216^ developed a Python script to query the MycoCosm genome database (https://mycocosm.JGI.doe.gov/mycocosm/home) for SMs targeting either the proteasome β6 subunit or the HMG-CoA reductase. This resulted in the identification of putatively novel inhibitors of HMG-CoA reductase in *Aspergillus* genomes.^216^ However, both aforementioned tools required bioinformatics skills for correct usage. FunARTS (“fungal bioactive compound resistant target seeker”) was then created by Yılmaz *et al.*,^217^ and made publicly available at https://funarts.ziemertlab.com as a user-friendly web tool. This workflow built on their bacterial version ARTS and extended their search to fungal genomes.^218,219^ FunARTS uses Hidden Markov Models to identify core genes of a BGC and co-localised known resistance genes to prioritise novel BGCs. It can then be linked to BiG-SCAPE^184^ to perform gene cluster network analysis.

### 4.4 Recent progress in machine learning for BGC prediction

Novel approaches using machine learning for BGC prediction have now seen the light with ClusterFinder^220^ or DeepBGC^181^ as prime examples. Specifically for fungi, a model using reinforcement learning was built and tested on *A. niger* and *A. nidulans*. The model, relying on protein domains and functional annotation, outperformed other software like fungiSMASH, TOUCAN and DeepBGC in cluster prediction.^221^ Other machine learning programs also focus on prediction of bioactivity. In antiSMASH, the core structure of polyketides or non-ribosomal peptides can be roughly predicted thanks to the integration of NRPSPredictor2 ^222^ which uses support vector machines, and methods from Minowa *et al.*^223^ and Starcevic *et al.*^224^ However, the accuracy of prediction remains subpar and based on bacterial-generated data. More recently, Walker *et al.*^225^ built a machine learning program to predict SM antibacterial, antifungal and cytotoxic activities directly from gene sequences. They achieved accuracy of up to 80% using the PFAM, MiBIG and Resistance Gene Identifier databases. As the model is trained prior to predictions, it can be applied to any well-curated database, including fungal ones. Finally, a fungi-specific platform for bioactivity prediction was created by Riedling *et al.*,^226^ but its performance is still lacking accuracy, as trained models were only able to reach scores of up to 68%. Bigger databases with well-curated data are crucial to advance the field of machine learning-based discovery of natural products, one of the main points of the extensive review by Mullowney *et al.*^227^ on the use of artificial intelligence in natural product discovery. Careful usage of those tools remains the golden rule and thorough reflection is needed when it comes to choosing algorithms. Towards the curation of better repositories for fungal genomes, Robey *et al.*^228^ constructed an atlas of 1037 fungal genomes with their respective biosynthetic content, paving the way for improved genome mining strategies.

### 4.5 Metabologenomics: a framework for natural product discovery

Metabologenomics is a concept that was first introduced in 2016 by the Kelleher lab.^229^ When dealing with large datasets housing hundreds if not thousands or different strains, the use of pattern-based genome mining coupled with molecular networking has proven to be game-changing. One significant study on this topic described the investigation of *Salinispora* genomes and enabled the characterisation of retimycin A, a quinomycin-like depsipeptide.^230^ Most recently, the NegMDF strategy was created to standardise this approach for the discovery of novel polyketides in bacteria.^231^ In this study, the authors employ a BGC-guided mass defect filtering (MDF) approach in parallel with negative mode MS scans to screen for novelty and use targeted MS/MS and NMR for validation. The MDF approach showed great advantages for the detection and prediction of bacterial PKSs as it does not rely on MS/MS fragmentation to infer product ions, offering limited bias towards abundant ions. Using this method, Liu *et al.*^231^ were able to identify novel polyketides in *Streptomyces cattleya* NRRL 8057, namely cattleyatetronates. Additional studies on bacteria and reviews on the subject attest of the rising interest in this approach.^162,229,232–239^ A community resource was created in 2021, the Paired Omics Data Platform, to offer scientists a database which facilitates the study of natural product biosynthesis based on both metabolomics and genomics data, with over 4800 genome-metabolome links with attached metadata.^240^ Recently, it was applied in 110 ascomycetes and was able to link more than 200 specialised metabolites to gene cluster families, within which the biosynthesis of pestalamides was uncovered.^241^ This workflow was then linked to bioactivity to further improve prioritisation of SM discovery, leading to the isolation of three novel stemphones, 19-acetylstemphones G, B and E.^242^ However, most studies using this approach focus on bacterial natural products, leaving fungal metabolites under-represented in this area of natural product research. More interest needs to be given to this highly promising approach for discovery.

## 5 Genetic manipulation in *Trichoderma* for the study of natural product biosynthesis

After prioritising BGCs using all aforementioned methods, experimental validation is necessary to link the novel natural products to the BGCs. This can be done using various strategies for fungal organisms, depending on their cultivability, genetic tractability and genetic engineering tools available for the fungal host. A decision tree to establish a workflow was designed in the review by Kjærbølling *et al.*^215^

### 5.1 Engineering in the host organism

If the fungal strain is cultivable and genetically tractable, investigation using the native host can be used and transformation protocols have been established for *Trichoderma* spp.^243–245^ Strategies to activate silent clusters through cultivation have already been detailed previously. Using genetic engineering approaches like transcription factor (TF) overexpression and promoter replacement or heterologous expression can also trigger and/or increase expression.

#### 5.1.1 Untargeted regulation of BGCs

Global regulators can be modulated for general activation of BGCs if no cluster-specific TF can be found. The concept was first applied to *Aspergillus* species, exemplified by the effects of LaeA on SM production including lovastatin, penicillin, and sterigmatocystin.^246^ In *Trichoderma*, the MAPkinases Tmk1 and Tmk3 were shown to impact expression of cellulase genes, as well as sorbicillinoid production.^247^ Focusing on *T. reesei* Rut-C-30, Yang *et al.*^248^ found that the TF *Ypr1* was involved in the regulation of cellulase production and secondary metabolism. Indeed, BGCs for specific polyketides and non-ribosomal peptides were found to be activated upon *Ypr1* gene deletion. Additionally, in *Trichoderma virens*, *Vel1* mutants displayed decreased levels of SM genes, including 3 NRPSs, 2 PKSs and abolished production of gliotoxin.^249^ A *Vel1* orthologue was later found in *T. reesei*,^250^*T. atroviride*^251^ and *T. asperellum*^252^ amongst others.^252^ Similarly, the LaeA protein has been shown to positively regulate specialised metabolism in *Trichoderma*. Overepxression of Talae1 in *T. afroharzianum* led to the discovery of two new sorbicillin-like polyketides, one of them showing strong antifungal activity against both *B. cinerea* and *F. oxysporum*.^253^ Similarly, in *T. longibrachiatum* SMF2, constitutive expression of Tllae1 doubled the production of peptaibols.^254^ In *T. atroviride*, it was found that LAE1 contributes to the formation of antifungal compounds active against *R. solani*, *B. cinerea* and *A. alternata*, as the overexpressing strain displayed improved inhibition against those fungal pathogens, linked to the expression of cell wall degrading enzymes and polyketide synthases but no specific compounds were characterised.^255^

#### 5.1.2 Targeted regulation of BGCs

However, manipulation of global regulators is well-known to be unpredictable and hard to control or reproduce. When cluster-specific TFs can be found, targeted manipulation can result in activation of the cluster. Most of the work on this topic has been done in *Aspergillus* or *Fusarium*. In *A. nidulans*, the structure and BGC for aspyridones A and B were identified by expressing the cluster-specific TF using an inducible promoter.^256,257^ In *F. fujikuroi*, the overexpression of the TF and PKS within the BGC led to the discovery of four new metabolites, namely fujikurins A–D.^258^ However, studies in *Trichoderma* have historically focused on improving cellulase production for industrial purposes.^248,259–262^ In the broader context of natural products, studies are scarcer. One example is in *T. afroharzianum* T-22 where overexpression of the tlnl TF in the tricholignan A BGC led to the overexpression of many novel specialised metabolites.^263^ When tested, tricholignan A (27) displayed plant growth promoting properties due to its redox activity.

In order to manipulate TFs effectively, the use of promoter engineering is needed. Reports of promoter engineering in actinomycetes and other bacteria are now abundant,^264^ but examples of implementations in *Trichoderma* are scarcer, with most studies focusing on *T. reesei* and overproduction of cellulases.^265–268^ In *Trichoderma*, a list of promoters and their use for overproduction of cellulase-encoding genes was established by Adnan *et al.*^267^ Most recently, a study in *Aspergillus nidulans* uncovered 93 promoter sequences from 454 TFs from transcriptome data and tested them for relative transcriptional strength using a single cell flow cytometry-based quantification method. From those, two strong promoters were chosen and introduced into *A. fumigatus* to drive transcription of the NRPS gene *Afpes1*. This led to the isolation and identification of fumiganins A and B.^269^ This work establishes a more comprehensive set of promoter sequences and applies them to other fungal species for natural product discovery. However, the use of promoter exchange is commonly labour intensive and requires a robust method for homologous recombination in the host.

Another approach involves the use of CRISPR/Cas technologies (Clustered Regularly Interspaced Short Palindormic Repeats).The clustered regularly interspaced short palindromic repeats and associated Cas9 nuclease (CRISPR-Cas9) have revolutionised the field of genetic engineering for its versatility and preciseness.^270^ In this system, the endonuclease Cas9 is guided by a single guide RNA (sgRNA) to a target locus in the genome and performs a double-strand break. The break can then be repaired *via* the Non-Homologous End Joining pathway which introduces mutations (insertions, deletions), or homologous recombination if provided with donor DNA. This is now well-established in model organisms such as *Escherichia coli*,^271^*Saccharomyces cerevisiae*,^272^ and *Streptomyces* spp.^273^ CRISPR-Cas9 systems have also been developed for filamentous fungi, including *Aspergillus* spp.,^274–276^*Fusarium* spp.^277–279^ and *Trichoderma* spp.^280^ A recent extensive review on the different tools available for CRISPR-mediated editing in filamentous fungi has been published by Woodcraft *et al.*,^281^ but specific studies of applications in *Trichoderma* spp. are scarce, with only two recent examples. Indeed, in a study by Wang *et al.*,^282^ researchers were able to activate the silent ilicicolin H (30) BGC in *T. reesei* using a new quinic acid-inducible Cas9. In the same species, Fang *et al.*^283^ were able to transform the 32.7 kb sorbicillinoids BGC, split into 10 fragments, into the *clr2* locus using CRISPR/Cas9 and in a single transformation step. However, no production of sorbicillinoids was observed due to the presence of three point mutations in the biosynthetic genes. Nevertheless, this method called simultaneous *in vivo* assembly and targeted genome integration of multiple DNA fragments (SATIMD), opens new avenues for the use of CRISPR/Cas9 in *Trichoderma* for the heterologous expression of BGCs from *Trichoderma* spp.^283^

### 5.2 Heterologous expression in model organisms

When the fungal host is not genetically tractable or cultivable, researchers will rely on heterologous expression. The choice of host is crucial and highly dependent on the nature of the cryptic BGC. *Escherichia coli* is a powerful host due to its fast growth, simple cultivation and easy and highly efficient transformation. It is generally used to express a single gene from the cluster of interest and coupled with *in vitro* experiments to study enzymatic structure and function.^285^ This was done in *T. atroviride* and led to the isolation of a new sesquiterpene alcohol trichobrasinelol (12). The sesquiterpene cyclase was cloned into *E. coli* BL21 and the expressed protein was purified and characterised for substrate specificity.^286^ However, the use of *E. coli* as a host for fungal BGCs usually shows limitations due to the presence of introns, codon bias, the lack of post-translational modifications, the potential toxicity of the products, and the availability of the precursors.^285^

*Saccharomyces cerevisiae* is one of the preferred hosts for the heterologous expression of fungal BGCs due to its low specialised metabolism background, its fast growth for a eukaryotic organism, its well-curated genetic engineering tools, its high homologous recombination rates, and its ability to correctly synthesise and fold fungal proteins, being a fungal organism.^287–289^ In the case of *Trichoderma* spp., *S. cerevisiae* has been used as heterologous host to reconstitute the biosynthetic pathway of several trichothecenes.^86,88,290^ Indeed, the final product trichodermol (13) was successfully biosynthesised heterologously in yeast using a codon optimised trichodiene synthase gene, coupled with a multicopy integration plasmid targeting the repetitive chromosomal rDNA.^290^ More recently, a heterologous expression platform (HEx) was developed to enable expression of fungal BGCs into *S. cerevisiae*.^291^ Using this platform, 41 BGCs from various ascomycetes, and basidiomycetes were integrated into yeast and of those, 54% produced specialised metabolites novel to yeast, including a PKS BGC from *T. virens*. This strategy enables high-throughput expression of a multitude of BGCs from various origins.

**Fig. 5:**
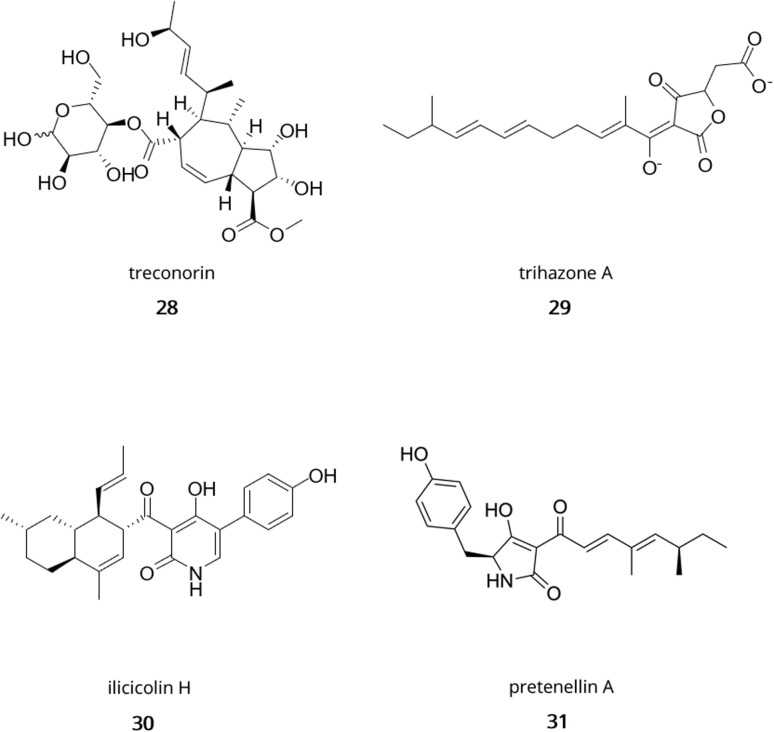
Examples of hybrid molecules from *Trichoderma*. Treconorin (28); trihazone A (29); ilicicolin H (30); pretenellin A (31).

However, for *Trichoderma* natural products, the use of filamentous fungi remains the most used, with *A. oryzae*,^292^*A. niger* (ATNT system),^293^*A. nidulans*^294^ and more recently *T. reesei*^295–297^ as heterologous hosts. Examples of studies using *A. oryzae* or *A. nidulans* as host have been described previously and can be found in the review by Shenouda *et al.*,^298^ with the ilicicolin H BGC as a recent case. In *A. niger*, a heterologous expression strain with altered NHEJ and altered pigmentation, has now been optimised for expression of long biosynthetic genes (over 20 kb) from ascomycetes, basidiomycetes and early diverging fungi.^299^ However, the main advantages of using *T. reesei* as a heterologous host for investigating *Trichoderma* BGCs is its phylogenetic closeness with other *Trichoderma* species and its ability to grow on cellulosic biomass, enabling the valorisation of waste material. This is the rationale behind the work by Shenouda *et al.*,^296^ where *T. reesei* was grown on peels from various fruits, used coffee grounds or barley straw to produce several specialised metabolites like pretenellin A (Fig. 5, 31) in a newly engineered strain, designed for SM production. This strain was provided a cleaner SM background by knocking out its sorbicillin BGC, and transformed with a vector containing the pretenellin A PKS-NRPS megasynthase with its *trans*-acting ER under the control of 2 constitutive promoters.^296^

However, heterologous expression can still prove challenging. This is exemplified by Hang *et al.*^300^ and their studies on the PKS Tv6-931 from *T. virens*. Their first attempt at heterologous expression of the PKS, as well as the entire BGC, failed to produce a new compound in *S. cerevisiae* and *A. nidulans*. They were only able to isolate new tetraketide products when a proper offloading substrate was added to the reaction. This shows that availability of substrates can impose limitations in various heterologous hosts.

## 6 Conclusions

**Fig. 6:**
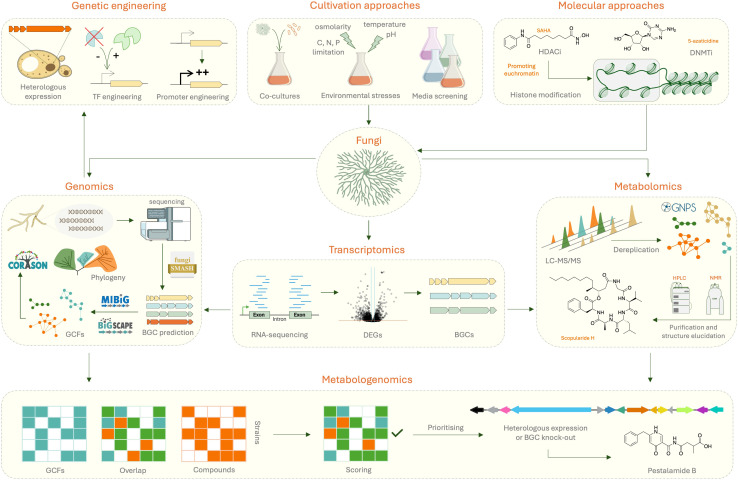
Strategies for natural product discovery in fungi. The OSMAC (“One Strain Many Compounds”) approach which includes modulating cultivation conditions or using molecular approaches like epigenetic modifications or ribosome engineering. Dereplication is essential to ensure novelty and is exemplified by the discovery of scopularide H from *Scopulariopsis* sp. CMB-F115 using GNPS.^284^ If the product can be detected, a combination of genomics, transcriptomics and metabolomics can be used to isolate the target SM, identify its structure, and link it to a gene cluster using several genome mining tools. This is the concept behind metabologenomics where the use of pattern-based genome mining coupled with molecular networking can provide insight into which clusters to prioritise for BGC-SM discovery. Validation of the link can then be done *via* a variety of different genetic engineering strategies such as TF or promoter engineering, heterologous expression or CRISPR/Cas technologies. This was exemplified by the discovery of pestalamide B along with its BGC from the native host *Aspergillus brasiliensis* and heterologously expressed in *A. nidulans*.^241^

To conclude, a combination of culture-based approaches and molecular approaches should be used to explore the biosynthetic potential of *Trichoderma* species, guided by genome mining strategies. The advances in this field and the increasingly available tools for genome engineering in *Trichoderma* and other filamentous fungi are tremendously helping discovery rates. Alongside, the development of tools for metabolomics are becoming indispensable for dereplication and prioritisation of compounds to prevent re-discovery of known SMs. Finally, an integrated platform combining omics strategies is now proving highly efficient in selecting cryptic BGCs to focus efforts on the discovery of new bioactive metabolites (Fig. 6).

## 8 Conflicts of interest

There are no conflicts to declare.

## Data Availability

No primary research results, software or code have been included and no new data were generated or analysed as part of this review.
